# The Rise and Impact of COVID-19 in India

**DOI:** 10.3389/fmed.2020.00250

**Published:** 2020-05-22

**Authors:** S. Udhaya Kumar, D. Thirumal Kumar, B. Prabhu Christopher, C. George Priya Doss

**Affiliations:** ^1^School of Biosciences and Technology, Vellore Institute of Technology, Vellore, India; ^2^VIT-BS, Vellore Institute of Technology, Vellore, India

**Keywords:** COVID-19, SARS-CoV-2, India, economy, safety measures

## Abstract

The coronavirus disease (COVID-19) pandemic, which originated in the city of Wuhan, China, has quickly spread to various countries, with many cases having been reported worldwide. As of May 8th, 2020, in India, 56,342 positive cases have been reported. India, with a population of more than 1.34 billion—the second largest population in the world—will have difficulty in controlling the transmission of severe acute respiratory syndrome coronavirus 2 among its population. Multiple strategies would be highly necessary to handle the current outbreak; these include computational modeling, statistical tools, and quantitative analyses to control the spread as well as the rapid development of a new treatment. The Ministry of Health and Family Welfare of India has raised awareness about the recent outbreak and has taken necessary actions to control the spread of COVID-19. The central and state governments are taking several measures and formulating several wartime protocols to achieve this goal. Moreover, the Indian government implemented a 55-days lockdown throughout the country that started on March 25th, 2020, to reduce the transmission of the virus. This outbreak is inextricably linked to the economy of the nation, as it has dramatically impeded industrial sectors because people worldwide are currently cautious about engaging in business in the affected regions.

## Current Scenario in India

**Figure 1:**
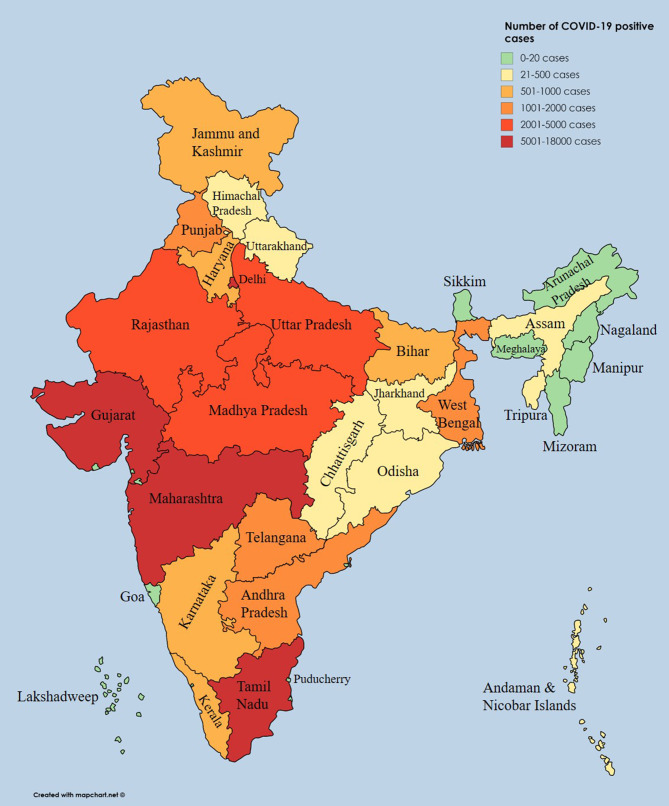
State-wise distribution of positive coronavirus disease cases displayed on an Indian geographical map.

Severe acute respiratory syndrome coronavirus 2 (SARS-CoV-2), which causes coronavirus disease (COVID-19), was first identified in December 2019 in Wuhan city, China, and later spread to many provinces in China. As of May 8th, 2020, the World Health Organization (WHO) had documented 3,759,967 positive COVID-19 cases, and the death toll attributed to COVID-19 had reached 259,474 worldwide (1). So far, more than 212 countries and territories have confirmed cases of SARS-CoV-2 infection. On January 30th, 2020, the WHO declared COVID-19 a Public Health Emergency of International Concern (2). The first SARS-CoV-2 positive case in India was reported in the state of Kerala on January 30th, 2020. Subsequently, the number of cases drastically rose. According to the press release by the Indian Council of Medical Research (ICMR) on May 8th, 2020, a total of 14,37,788 suspected samples had been sent to the National Institute of Virology (NIV), Pune, and a related testing laboratory (3). Among them, 56,342 cases tested positive for SARS-CoV-2 (4). A state-wise distribution of positive cases until May 8th, 2020, is listed in Table 1, and the cases have been depicted on an Indian map (Figure 1). Nearly 197,192 Indians have recently been repatriated from affected regions, and more than 1,393,301 passengers have been screened for SARS-CoV-2 at Indian airports (5), with 111 positive cases observed among foreign nationals (4, 5). As of May 8th, 2020, Maharashtra, Delhi, and Gujarat states were reported to be hotspots for COVID-19 with 17,974, 5,980, and 7,012 confirmed cases, respectively. To date, 16,540 patients have recovered, and 1,886 deaths have been reported in India (5). To impose social distancing, the “Janata curfew” (14-h lockdown) was ordered on March 22nd, 2020. A further lockdown was initiated for 21 days, starting on March 25th, 2020, and the same was extended until May 3rd, 2020, but, owing to an increasing number of positive cases, the lockdown has been extended for the third time until May 17th, 2020 (6). Currently, out of 32 states and eight union territories in India, 26 states and six union territories have reported COVID-19 cases. Additionally, the health ministry has identified 130 districts as hotspot zones or red zones, 284 as orange zones (with few SARS-CoV-2 infections), and 319 as green zones (no SARS-CoV-2 infection) as of May 4th, 2020. These hotspot districts have been identified to report more than 80% of the cases across the nation. Nineteen districts in Uttar Pradesh are identified as hotspot districts, and this was followed by 14 and 12 districts in Maharashtra and Tamil Nadu, respectively (7). The complete lockdown was implemented in these containment zones to stop/limit community transmission (5). As of May 8th, 2020, 310 government laboratories and 111 private laboratories across the country were involved in SARS-CoV-2 testing. As per ICMR report, 14,37,788 samples were tested till date, which is 1.04 per thousand people (3).

**Table 1:** Current status of reported positive coronavirus disease cases in India (State-wise).

**S. no**.	**State name/UT**	Confirmed cases^*^	**Cured/discharged/migrated**	**Death**
1	Andhra Pradesh	1,847	780	38
2	Andaman and Nicobar Islands	33	33	0
3	Arunachal Pradesh	1	1	0
4	Assam	54	34	1
5	Bihar	550	246	5
6	Chandigarh	135	21	1
7	Chhattisgarh	59	38	0
8	Delhi	5,980	1,931	66
9	Goa	7	7	0
10	Gujarat	7,012	1,709	425
11	Haryana	625	260	7
12	Himachal Pradesh	46	38	2
13	Jammu and Kashmir	793	335	9
14	Jharkhand	132	41	3
15	Karnataka	705	366	30
16	Kerala	503	474	4
17	Ladakh	42	17	0
18	Madhya Pradesh	3,252	1,231	193
19	Maharashtra	17,974	3,301	694
20	Manipur	2	2	0
21	Meghalaya	12	10	1
22	Mizoram	1	0	0
23	Odisha	219	62	2
24	Puducherry	9	6	0
25	Punjab	1,644	149	28
26	Rajasthan	3,427	1,596	97
27	Tamil Nadu	5,409	1,547	37
28	Telengana	1,123	650	29
29	Tripura	65	2	0
30	Uttarakhand	61	39	1
31	Uttar Pradesh	3,071	1,250	62
32	West Bengal	1,548	364	151
**Total number of positive cases reported in India**		56,342^*^	**16,540**	**1,886**

*Data source: available from Ministry of Health and Family Welfare, India (https://www.mohfw.gov.in/)*.

* *Positive coronavirus disease cases including 111 foreign Nationals and cases are being increased; UT, Union Territories*.

## COVID-19 and Previous Coronavirus Outbreaks

The recent outbreak of COVID-19 in several countries is similar to the previous outbreaks of SARS and Middle East respiratory syndrome (MERS) that emerged in 2003 and 2012 in China and Saudi Arabia, respectively (8–10). Coronavirus is responsible for both SARS and COVID-19 diseases; they affect the respiratory tract and cause major disease outbreaks worldwide. SARS is caused by SARS-CoV, whereas SARS-CoV-2 causes COVID-19. So far, there is no particular treatment available to treat SARS or COVID-19. In the current search for a COVID-19 cure, there is some evidence that point to SARS-CoV-2 being similar to human coronavirus HKU1 and 229E strains (11, 12) even though they are new coronavirus family members. These reports suggest that humans do not have immunity to this virus, allowing its easy and rapid spread among human populations through contact with an infected person. SARS-CoV-2 is more transmissible than SARS-CoV. The two possible reasons could be (i) the viral load (quantity of virus) tends to be relatively higher in COVID-19-positive patients, especially in the nose and throat immediately after they develop symptoms, and (ii) the binding affinity of SARS-CoV-2 to host cell receptors is higher than that of SARS-CoV (13, 14). The other comparisons between SARS and COVID-19 are tabulated in Table 2, and references for the same are provided here (1, 15, 16).

**Table 2:** Differences between coronavirus disease and severe acute respiratory syndrome.

	**Severe acute respiratory syndrome**	**Coronavirus disease**
Preliminary key symptoms	Fever, respiratory symptoms, cough, malaise	Cough, fever, and shortness of breath
First exposure	November 2002	December 2019
First detected location	Guangdong Province, China	Wuhan, China
Global cases	8,098 cases	3,759,967– (Until May 8th, 2020)
Number of countries infected	26	212 including territories
Global deaths	774	259,474 (Until May 8th, 2020)
Mortality rate	15%	3–4%
Mode of transmission	Respiratory droplets and contaminated surfaces	Respiratory droplets along with feces and other bodily discharges
Most affected age groups	≥ 60 (55% mortality rate)	People of all ages are affected. Older people and people with medical illness, such as asthma, diabetes, and heart disease, succumb more easily to severe illness
Treatment	No effective treatment or cure. Antivirals and steroids showed promising results for few patients	No effective treatment or cure. Supportive care, pain relievers, and fever reducers can alleviate symptoms. Few antibiotics and antivirals are administered in drug repurposing way to help with recovery
End of pandemic	July 2003	Still active

## Impact of COVID-19 in India and the Global Economy

As per the official government guidelines, India is making preparations against the COVID-19 outbreak, and avoiding specific crisis actions or not understating its importance will have extremely severe implications. All the neighboring countries of India have reported positive COVID-19 cases. To protect against the deadly virus, the Indian government have taken necessary and strict measures, including establishing health check posts between the national borders to test whether people entering the country have the virus (17). Different countries have introduced rescue efforts and surveillance measures for citizens wishing to return from China. The lesson learned from the SARS outbreak was first that the lack of clarity and information about SARS weakened China's global standing and hampered its economic growth (10, 18–20). The outbreak of SARS in China was catastrophic and has led to changes in health care and medical systems (18, 20). Compared with China, the ability of India to counter a pandemic seems to be much lower. A recent study reported that affected family members had not visit the Wuhan market in China, suggesting that SARS-CoV-2 may spread without manifesting symptoms (21). Researchers believe that this phenomenon is normal for many viruses. India, with a population of more than 1.34 billion—the second largest population in the world—will have difficulty treating severe COVID-19 cases because the country has only 49,000 ventilators, which is a minimal amount. If the number of COVID-19 cases increases in the nation, it would be a catastrophe for India (22). It would be difficult to identify sources of infection and those who come in contact with them. This would necessitate multiple strategies to handle the outbreak, including computational modeling as well as statistical and quantitative analyses, to rapidly develop new vaccines and drug treatments. With such a vast population, India's medical system is grossly inadequate. A study has shown that, owing to inadequate medical care systems, nearly 1 million people die every year in India (23). India is also engaged in trading with its nearby countries, such as Bangladesh, Bhutan, Pakistan, Myanmar, China, and Nepal. During the financial year 2017–18 (FY2017–18), Indian regional trade amounted to nearly $12 billion, accounting for only 1.56% of its total global trade value of $769 billion. The outbreak of such viruses and their transmission would significantly affect the Indian economy. The outbreak in China could profoundly affect the Indian economy, especially in the sectors of electronics, pharmaceuticals, and logistics operations, as trade ports with China are currently closed. This was further supported by the statement by Suyash Choudhary, Head—Fixed Income, IDFC AMC, stating that GDP might decrease owing to COVID-19 (24).

Economists assume that the impact of COVID-19 on the economy will be high and negative when compared with the SARS impact during 2003. For instance, it has been estimated that the number of tourists arriving in China was much higher than that of tourists who traveled during the season when SARS emerged in 2003. This shows that COVID-19 has an effect on the tourism industry. It has been estimated that, for SARS, there was a 57 and 45% decline in yearly rail passenger and road passenger traffic, respectively (25). Moreover, when compared with the world economy 15 years ago, world economies are currently much more inter-related. It has been estimated that COVID-19 will hurt emerging market currencies and also impact oil prices (26–28). From the retail industry's perspective, consumer savings seem to be high. This might have an adverse effect on consumption rates, as all supply chains are likely to be affected, which in turn would have its impact on supply when compared with the demand of various necessary product items (29). This clearly proves that, based on the estimated losses due to the effect of SARS on tourism (retail sales lost around USD 12–18 billion and USD 30–100 billion was lost at a global macroeconomic level), we cannot estimate the impact of COVID-19 at this point. This will be possible only when the spread of COVID-19 is fully controlled. Until that time, any estimates will be rather ambiguous and imprecise (19). The OECD Interim economic assessment has provided briefing reports highlighting the role of China in the global supply chain and commodity markets. Japan, South Korea, and Australia are the countries that are most susceptible to adverse effects, as they have close ties with China. It has been estimated that there has been a 20% decline in car sales, which was 10% of the monthly decline in China during January 2020. This shows that even industrial production has been affected by COVID-19. So far, several factors have thus been identified as having a major economic impact: labor mobility, lack of working hours, interruptions in the global supply chain, less consumption, and tourism, and less demand in the commodity market at a global level (30), which in turn need to be adequately analyzed by industry type. Corporate leaders need to prioritize the supply chain and product line economy trends via demand from the consumer end. Amidst several debates on sustainable economy before the COVID-19 impact, it has now been estimated that India's GDP by the International Monetary Fund has been cut down to 1.9% from 5.8% for the FY21. The financial crisis that has emerged owing to the worldwide lockdown reflects its adverse effect on several industries and the global supply chain, which has resulted in the GDP dropping to 4.2% for FY20, which was previously estimated at 4.8%. Nevertheless, it has been roughly estimated that India and China will be experiencing considerable positive growth among other major economies (31).

## Preparations and Preventive Measures in India

An easy way to decrease SARS-CoV-2 infection rates is to avoid virus exposure. People from India should avoid traveling to countries highly affected with the virus, practice proper hygiene, and avoid consuming food that is not home cooked. Necessary preventive measures, such as wearing a mask, regular hand washing, and avoiding direct contact with infected persons, should also be practiced. The Ministry of Health and Family Welfare (MOHFW), India, has raised awareness about the recent outbreak and taken necessary action to control COVID-19. Besides, the MOHFW has created a 24 h/7 days-a-week disease alert helpline (+91-11-23978046 and 1800-180-1104) and policy guidelines on surveillance, clinical management, infection prevention and control, sample collection, transportation, and discharging suspected or confirmed cases (3, 5). Those who traveled from China, or other countries, and exhibited symptoms, including fever, difficulty in breathing, sore throat, cough, and breathlessness, were asked to visit the nearest hospital for a health check-up. Officials from seven different airports, including Chennai, Cochin, New Delhi, Kolkata, Hyderabad, and Bengaluru, have been ordered to screen and monitor Indian travelers from China and other affected countries. In addition, a travel advisory was released to request the cessation of travel to affected countries, and anyone with a travel history that has included China since January 15th, 2020, would be quarantined. A centralized control room has been set up by the Delhi government at the Directorate General of Health Services, and 11 other districts have done the same. India has implemented COVID-19 travel advisory for intra- and inter-passenger aircraft restrictions. More information on additional travel advisory can be accessed with the provided link (https://www.mohfw.gov.in/pdf/Traveladvisory.pdf).

India is known for its traditional medicines in the form of AYUSH (Ayurvedic, Yoga and Naturopathy, Unani, Siddha, and Homeopathy). The polyherbal powder NilavembuKudineer showed promising effects against dengue and chikungunya fevers in the past (32). With the outbreak of COVID-19, the ministry of AYUSH has released a press note “Advisory for Coronavirus,” mentioning useful medications to improve the immunity of the individuals (33). Currently, according to the ICMR guidelines, doctors prescribe a combination of Lopinavir and Ritonavir for severe COVID-19 cases and hydroxychloroquine for prophylaxis of SARS-CoV-2 infection (34, 35). In collaboration with the WHO, ICMR will conduct a therapeutic trial for COVID-19 in India (3). The ICMR recommends using the US-FDA-approved closed real-time RT-PCR systems, such as GeneXpert and Roche COBAS-6800/8800, which are used to diagnose chronic myeloid leukemia and melanoma, respectively (36). In addition, the TruenatTM beta CoV test on the TruelabTM workstation validated by the ICMR is recommended as a screening test. All positive results obtained on this platform need to be confirmed by confirmatory assays for SARS-CoV-2. All negative results do not require further testing. Antibody-based rapid tests were validated at NIV, Pune, and found to be satisfactory; the rapid test kits are as follows: (i) SARS-CoV-2 Antibody test (Lateral flow method): Guangzhou Wondfo Biotech, Mylan Laboratories Limited (CE-IVD); (ii) COVID-19 IgM&IgG Rapid Test: BioMedomics (CE-IVD); (iii) COVID-19 IgM/IgG Antibody Rapid Test: Zhuhai Livzon Diagnostics (CEIVD); (iv) New coronavirus (COVID-19) IgG/IgM Rapid Test: Voxtur Bio Ltd, India; (v) COVID-19 IgM/IgG antibody detection card test: VANGUARD Diagnostics, India; (vi) MakesureCOVID-19 Rapid test: HLL Lifecare Limited, India; and (vii) YHLO SARS-CoV-2 IgM and IgG detection kit (additional equipment required): CPC, Diagnostics. As a step further, on the technological aspect, the Union Health Ministry has launched a mobile application called “AarogyaSetu” that works both on android and iOS mobile phones. This application constructs a user database for establishing an awareness network that can alert people and governments about possible COVID-19 victims (37).

## Future Perspectives

Infections caused by these viruses are an enormous global health threat. They are a major cause of death and have adverse socio-economic effects that are continually exacerbated. Therefore, potential treatment initiatives and approaches need to be developed. First, India is taking necessary preventive measures to reduce viral transmission. Second, ICMR and the Ministry of AYUSH provided guidelines to use conventional preventive and treatment strategies to increase immunity against COVID-19 (3, 38). These guidelines could help reduce the severity of the viral infection in elderly patients and increase life expectancy (39). The recent report from the director of ICMR mentioned that India would undergo randomized controlled trials using convalescent plasma of completely recovered COVID-19 patients. Convalescent plasma therapy is highly recommended, as it has provided moderate success with SARS and MERS (40); this has been rolled out in 20 health centers and will be increased this month (May 2020) (3). India has expertise in specialized medical/pharmaceutical industries with production facilities, and the government has established fast-tracking research to develop rapid diagnostic test kits and vaccines at low cost (41). In addition, the Serum Institute of India started developing a vaccine against SARS-CoV-2 infection (42). Until we obtain an appropriate vaccine, it is highly recommended that we screen the red zoned areas to stop further transmission of the virus. Medical college doctors in Kerala, India, implemented the low-cost WISK (Walk-in Sample Kiosk) to collect samples without direct exposure or contact (43, 44). After Kerala, The Defense Research and Development Organization (DRDO) developed walk-in kiosks to collect COVID-19 samples and named these as COVID-19 Sample Collection Kiosk (COVSACK) (45). After the swab collection, the testing of SARS-CoV-2 can be achieved with the existing diagnostic facility in India. This facility can be used for massive screening or at least in the red zoned areas without the need for personal protective equipment kits (43, 45). India has attempted to broaden its research facilities and shift toward testing the mass population, as recommended by medical experts in India and worldwide (46).

## Data Availability Statement

Publicly available datasets were analyzed in this study. This data can be found here: https://www.mohfw.gov.in/ and https://www.icmr.gov.in/.

## Author Contributions

SK, DK, and CD were involved in the design of the study and the acquisition, analysis, interpretation of the data, and drafting the manuscript. BC was involved in the interpretation of the data. CD supervised the entire study. The manuscript was reviewed and approved by all the authors.

## Conflict of Interest

The authors declare that the research was conducted in the absence of any commercial or financial relationships that could be construed as a potential conflict of interest.
